# Therapeutic Management of LDL-C: Efficacy and Economic Impact Assessment

**DOI:** 10.3390/jcdd12050196

**Published:** 2025-05-20

**Authors:** Abdallah Elshafeey

**Affiliations:** Department of Medicine, Johns Hopkins Hospital, Baltimore, MD 21287, USA; aelshaf1@jhmi.edu

**Keywords:** atherosclerotic cardiovascular disease, dyslipidemia, LDL-C, economic impact

## Abstract

Cardiovascular disease (CVD) is one of the largest global disease burdens. Despite guidelines and recommendations and the proven advantages of lipid-lowering therapies (LLTs) in preventing CVD, achieving treatment targets remains disappointing. A key barrier to optimal LLT is therapy discontinuation. To be widely adopted in clinical practice, new lipid-lowering therapies must both prevent major adverse cardiovascular events (MACEs) and exhibit cost effectiveness to ensure widespread utilization by patients, physicians, and insurers. While non-statin LLTs have shown cardiovascular value, their cost effectiveness is controversial. This review highlights the LLTs that are currently widely adopted and summarizes the available evidence on their cost effectiveness.

## 1. Introduction

CVD is one of the largest global disease burdens [1]. Atherosclerotic cardiovascular disease (ASCVD) and, subsequent, acute coronary syndromes (ACSs) are among the most significant global health concerns, due to their high prevalence, mortality rates, and the associated costs. The pathogenesis of atherosclerosis involves lipoproteins, mostly low-density lipoprotein (LDL) carrying apolipoprotein B, entering and accumulating in the subendothelial space, triggering an inflammatory response that leads to the accumulation of atherosclerotic plaques. These plaques eventually rupture, causing myocardial infarction [2,3]. ASCVD can be modified and prevented by lipid-lowering drugs. There is a significant and linear association between the level of LDL-C and the risk of ASCVD, which has been repeatedly established [4].

The key challenge is not whether decreasing the LDL-C concentration lowers CVD risk, as evidence consistently shows a linear and predictable risk reduction. Instead, the main concern lies in medication adherence, tolerability, and the societal cost of the treatment, which may influence the anticipated benefits of LDL-C reduction. This review aims to take a look at the lipid-lowering therapies (LLTs) that are currently widely adopted and summarizes the available evidence on their cost effectiveness.

## 2. Lipid-Lowering Drugs

Table 1 shows different studies on LCL-C-lowering treatments in various populations.

### 2.1. Statins

**Figure 1:**
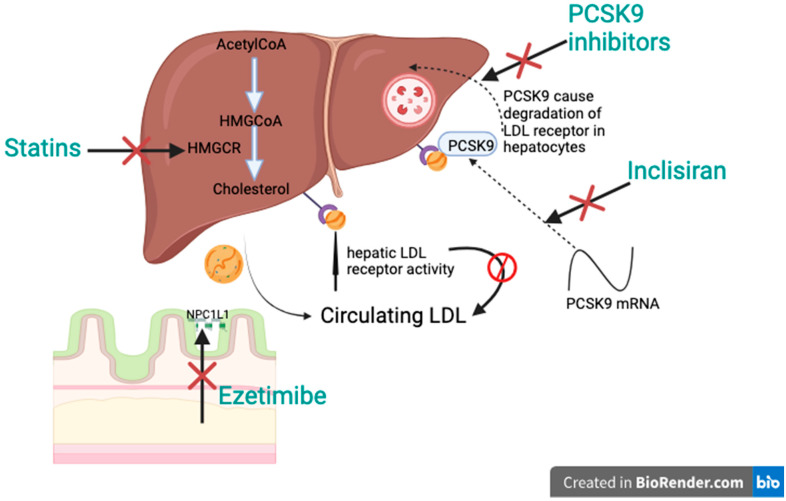
Additive effect of different therapies.

Statins are widely regarded as the pillar of lipid-lowering therapy, primarily due to their effectiveness, affordability, and tolerability. They function by inhibiting the enzyme 3-hydroxy-3-methylglutaryl-coenzyme A reductase (HMGCR), a key regulator in the biosynthesis of the cholesterol pathway. This inhibition decreases hepatic cholesterol production and increases hepatic LDL receptor activity, causing a significant decrease in circulating LDL-C levels (Figure 1) [26]. Statins are classified into low-, moderate-, and high-intensity therapies, according to their expected LDL-C-lowering capabilities. The intensity of the treatment is tailored to the patient’s baseline and target LDL-C level, taking into account their CVD risk profile and potential adverse effects at higher doses. For high-risk individuals, the principle of “lower is better” underlines the use of maximum tolerated doses.

Statins are recommended across various populations for both the primary and secondary prevention of CVD. High doses of statins, such as atorvastatin, simvastatin, and rosuvastatin, are advised for patients with heterozygous familial hypercholesterolemia [27]. Statins reduce non-fatal myocardial infarctions (MIs) and strokes in individuals aged 66–75, although their effect on all-cause mortality in the short term remains limited. Evidence supports statin therapy for elderly people with a background of stroke or MI to prevent recurrent cardiovascular events. Trials, such as PROSPER, demonstrated a reduction in non-fatal MIs and coronary deaths, among patients aged 70–82, treated with pravastatin [28]. Other studies, like the SAGE trial and the Heart Protection Study, have highlighted the role of statins in reducing major coronary events, without significant differences across age groups [29]. Statins also exhibit immediate protective effects in regard to ACSs, attributed to their anti-inflammatory and pleiotropic properties, beyond LDL-C lowering. Trials, such as MIRACL, PROVE IT-TIMI 22, and A-to-Z, revealed that initiating statin therapy shortly after an ACS event significantly reduced MACEs and markers of myocardial injury. Despite the extensive benefits, achieving LDL-C goals, particularly in ACS patients, often requires high-intensity statins and may take weeks to months of treatment. Furthermore, approximately 20% of ACS survivors experience recurrent ischemic events within two years. The early initiation and adherence to high-dose statins remain critical for optimizing outcomes, highlighting the importance of statin therapy in regard to both the acute and long-term management of CVD [30,31,32,33]. The American Diabetes Association (ADA) endorses statins for diabetic patients aged 40 and above with one or more CVD risk factors, regardless of their baseline lipid levels. They decrease the relative risk of coronary heart disease in patients with diabetes by approximately one third [34,35,36]. A cohort study, including 8937 subjects, investigated the optimum statin therapy in terms of intensity, duration, and LDL-C level on MACEs among T2D patients in a real-world setting. Moderate and high statin intensity, longer treatment duration, and lower accomplished LDL-C was associated with a lower risk of MACEs. An assessment of all three factors on cardiovascular risk (log likelihood) showed that the duration of statin treatment is more important than the level of LDL-C achieved or the statin intensity on CVD, highlighting the importance of patient adherence and proposing a “longer is better” concept rather than “lower is better” [37]. A meta-analysis comparing the effectiveness of atorvastatin, rosuvastatin, and simvastatin, over an average treatment period of 12 weeks, on non-HDL-C, as the primary target, and LDL-C, total cholesterol, and cardiovascular events, such as non-fatal MI, non-fatal stroke, and death due to cardiovascular disease, as secondary targets, was conducted involving 20,193 diabetic patients. Rosuvastatin greatly lowered the level of non-HDL-C at high- (−2.31 mmol/L) and moderate-intensity doses (2.27 mmol/L), followed by simvastatin (−2.26) and atorvastatin (2.20) at high-intensity doses, compared to the placebo. High-intensity doses of atorvastatin were the most efficient for patients at high risk of MACEs [38].

Statins, while generally safe, may occasionally cause muscle-related adverse events that range from mild myopathy to severe rhabdomyolysis. Statins exhibit a high tolerability profile, with no significant differences in adverse event-related discontinuations between active treatment and placebo groups in randomized controlled trials (RCTs). Muscle aches without significant CK elevation are frequently reported in observational studies, with an incidence of 10–20% [39,40]. However, placebo-controlled trials attribute such symptoms to statins in only about 1 in 15 cases. This discrepancy is likely because of the “nocebo” or “drucebo” effect, where negative expectations about the drug play a role in the perceived symptoms [41,42]. Despite this, long-term adherence remains a challenge due to misconceptions about tolerability, particularly regarding muscle symptoms. Adherence to statin therapy can be improved through several approaches, like switching to agents like pravastatin, which may reduce adverse effects; lowering the dose or using alternate-day dosing can enhance tolerability; and addressing misconceptions and providing evidence-based reassurance, which can mitigate the nocebo effect. A percentage of patients, reaching up to 70%, who were deemed statin intolerant, may tolerate the therapy when these approaches are applied [43,44].

### 2.2. Ezetimibe

Ezetimibe is a Niemann-Pick C1-Like 1 (NPC1L1) inhibitor that blocks cholesterol absorption at the small intestinal brush border. By decreasing the transfer of cholesterol via chylomicrons to the liver, it lowers hepatic cholesterol stores. This triggers an increase in LDL receptor expression, enhancing hepatic LDL uptake and reducing plasma LDL-C levels (Figure 1) [45]. Large cardiovascular outcome studies have shown a safety and tolerability profile for ezetimibe that was comparable to the placebo. It reduces LDL-C by approximately 19–20% when used as a monotherapy and it provides an extra LDL-C decrease of 15–23% when combined with statins, depending on the intensity of statin therapy.

In a 3-year, South Korean, randomized, controlled, non-inferiority trial (RACING trial) conducted on 3780 atherosclerotic cardiovascular disease patients, 1894 patients received 10 mg of rosuvastatin with 10 mg of ezetimibe as a combination therapy, and 1886 patients received 20 mg of rosuvastatin as a high-intensity statin single therapy. The primary endpoints were cardiovascular death, due to MI, stroke, sudden cardiac death, cardiovascular procedures, heart failure, cardiovascular hemorrhage, and any death due to a cardiovascular reason; MACEs, including coronary or peripheral revascularization or hospitalization; or non-fatal stroke, within the study period. The study showed that the moderate-dose dual treatment was non-inferior to the high-dose statin for the study period, with a higher amount of patients with a percentage of their LDL cholesterol levels lower than 70 mg/dL and a lower discontinuation rate or dose reduction due to intolerance [5]. A meta-analysis studied the safety and efficacy of statins at high intensity compared to a low–moderate-intensity statin/ezetimibe dual treatment in individuals with atherosclerotic cardiovascular disease. Combined low–moderate-intensity statins and ezetimibe substantially decreased the LDL-C, but there was no significant difference in the total cholesterol, HDL-C, triglyceride, Apo A1 high-sensitivity C-reactive protein, or Apo B. The difference in the liver safety of both treatments was not significant, as depicted by the markers, aspartate aminotransferase and alanine aminotransferase; however, there was a significant difference in creatine phosphokinase [6]. A comparative cohort, Korean, observational study on the preventive effect of combined treatment with high-intensity and low- or moderate-intensity statins with ezetimibe, on CVD and all-cause death in people without prior cardiovascular disease, was conducted. Moderate-intensity statin together with ezetimibe significantly decreased the risk of a composite outcome, individual MIs and stroke, with a HR of 0.84, 0.81, and 0.78, respectively. No difference was observed in all-cause mortality in both groups. On the other hand, low-intensity statins together with ezetimibe decreased the risk of a composite outcome, with no difference in the individual outcomes. A limitation of this study is the possible existence of residual bias, as well as the limited ability to interpret casual relationships, due to the observational nature of the study [7]. An observational, retrospective, population-based, cohort study was conducted between 2013 and 2019 to compare the outcome of high-intensity and moderate-intensity statins combined with ezetimibe in ACS patients after a PCI. In the high-intensity statin subjects, the primary outcome (ischemic stroke, MIs, and all-cause mortality) was 4.73 events/100 person/year, and in the moderate-intensity statin/ezetimibe patients, it was 4.29 events/100 person/year. As for the risk of myocardial infarction, no difference was observed in regard to both treatments. However, the risk of stroke, HR 0.83, and all-cause mortality, HR 0.79, were lower in the group taking the moderate-intensity statins/ezetimibe. The dual-therapy patients exhibited higher compliance, 67.8% versus 50.0% in the group taking high-intensity statins, (*p* < 0.001) for 90 days. A drawback of this study is the absence of laboratory data, which makes it unclear whether the combination therapy reduced the LDL levels more effectively than high-intensity statins alone [8]. In a large observational study, including 45,501 patients who underwent a PCI at a follow-up of 2.7 years (median), it was found that using a combination treatment of moderate-intensity statins and ezetimibe was similar to high-intensity statin therapy in terms of the long-term risk and the decrease in MACEs. However, combination treatment was correlated with a substantial decrease in the occurrence of new-onset DM (12.5% in the case of monotherapy and 10.7% in the case of combination therapy). This study was unable to determine the rationale behind the use of either combination therapy or monotherapy. Compared to patients undergoing combination therapy, those undergoing high-intensity statin monotherapy may have less severe risk profiles for tolerating high-intensity stains. Despite the worse profiles of the patients, the ezetimibe combination treatment demonstrated comparable efficacy to monotherapy; therefore, this bias is unlikely to account for the results [9]. A meta-analysis involving 20,291 patients assessed the incorporation of ezetimibe and high-intensity statins at the time of ACS. The addition of ezetimibe significantly reduced LDL-C at 7 days (−19.55 mg/dL), 1 month (−24.67 mg/dL), 3 months (−18.01 mg/dL), and 10–12 months (−16.90 mg/dL) of treatment. It also significantly reduced the total cholesterol at 7 days (−21.05 mg/dL), 1 month (−25.56 mg/dL), 3 months (−22.54 mg/dL), and 12 months (−19.68 mg/dL) of treatment. Death due to any reason, non-fatal stroke, ACS, ischemic stroke, and non-fatal MIs were substantially decreased in subjects taking ezetimibe. The significant decrease in LDL-C led to a substantial decrease in major ACSs, ischemic stroke, non-fatal stroke, the risk of death from any cause, and non-fatal MIs [10]. An open-label, Korean, phase IV, multicenter, randomized controlled trial, comparing intensive rosuvastatin therapy, 20 mg, with rosuvastatin and ezetimibe combination therapy, 10/10, in ASCVD patients, in a 1:1 manner, was conducted. After treatment for 12 and 24 weeks, the dual treatment significantly decreased LDL-C compared to the high-intensity statin therapy (−22.9% for the combination therapy, −15.6% for the monotherapy, after 12 weeks; and −24.2% for the combination therapy, −12.9% for the monotherapy, after 24 weeks). Interestingly, there was no significant difference in the occurrence of the total range of side effects between both groups. Despite the substantial reduction in the level of LDL-C using the combination therapy, this study lacks an assessment of the clinical outcomes and cardiovascular events [11]. A Korean, phase III, multicenter, double-blind, randomized study was conducted to compare pitavastatin/ezetimibe dual treatment with pitavastatin in primary hypercholesterolemia patients. The combination treatment significantly decreased the LDL-C levels by >50%, with no significant difference in the tolerability and safety of the treatment in both groups [12]. A meta-analysis on data, including eighteen articles, was conducted to compare the effects of a dual treatment of low/moderate-intensity statins together with ezetimibe against statins at a high intensity on lipid parameters and hs-CRP. The low/moderate-intensity statins plus ezetimibe significantly decreased the LDL-C, TC, triglyceride, and hs-CRP versus the high-intensity statins. The statin single treatment significantly increased the AST and CK; however, there was no significant difference in the ALT between the two groups [46]. A meta-analysis comprising 3105 patients from 14 studies was conducted to compare the effect of ezetimibe/statin dual treatment (50.18% of participants) with double-dose statin monotherapy (49.82% of participants) in high cardiovascular risk patients. The dual treatment substantially decreased the LDL-C, TG, TC, and non-HDL-C concentrations. The atorvastatin and ezetimibe dual treatment caused more of a reduction in the non-HDL-C and TC concentrations versus 40 mg of atorvastatin; however, the difference between double-dose rosuvastatin and the ezetimibe/rosuvastatin dual treatment was not significant in regard to decreasing the LDL-C, TG, non-HDL-C, and TC concentrations [47].

### 2.3. Proprotein Convertase Subtilisin/Kexin Type 9 (PCSK9) Inhibitors

Proprotein convertase subtilisin/kexin type 9 (PCSK9) inhibitors are a transformative therapy for patients with high cardiovascular risk or refractory hyperlipidemia, providing substantial LDL-C reduction and improving cardiovascular outcomes. PCSK9 is a liver-produced protease that regulates LDL-C by promoting the degradation of LDL receptors in hepatocytes. By binding to LDL receptors, PCSK9 leads to their internalization and lysosomal destruction, reducing the liver’s capacity to eliminate LDL-C from the bloodstream. Inhibiting PCSK9 prevents receptor degeneration, allowing receptors to recycle to the surface of the hepatocyte, thereby lowering serum LDL-C levels (Figure 1). Evolocumab and alirocumab are fully humanized antibodies, given subcutaneously, every 2 or 4 weeks. Both drugs lower LDL-C by almost 60% when combined with the maximum tolerated statin treatment and have shown a favorable safety profile in large phase 3 trials [48,49].

The FOURIER and ODYSSEY trials showed a 15% decrease in MACEs over 2–3 years. Lipoprotein(a) levels are linked to cardiovascular events. The ODYSSEY outcomes trial analyzed the effect of alirocumab on lipoprotein(a) and LDL-C levels in 18,924 patients with a recent ACS. Over a follow-up of 2.8 years, alirocumab substantially decreased lipoprotein(a) by 5.0 mg/dL and adjusted LDL-C by 51.1 mg/dL, leading to a 15% reduction in MACEs. Reductions in both lipoprotein(a) and LDL-C independently projected a lower MACE risk, emphasizing lipoprotein(a) as an independent therapeutic target after ACS [13]. A retrospective cohort study, involving 1564 ACS Chinese subjects who underwent a PCI, studied the effect of the initiation of evolocumab on LDL-C levels, recurrent ischemic cardiovascular events, and safety for 18 months. The study incorporated individuals with high LDL-C (≥3.2 mmol/L without statin treatment, ≥2.3 mmol/L after taking statin at a low- or moderate-intensity or ≥1.8 mmol/L after taking high-intensity statin treatment for ≥4 weeks). The addition of evolocumab to statins decreased LDL-C by 42.48%. It also reduced the primary outcome; however, there was no significant difference in the safety of the treatments. Given the study’s retrospective design, selection bias might be present [14]. A meta-analysis, including 54,311 patients, evaluated the safety and efficacy of PCSK9 inhibitors for secondary prevention purposes in high-risk cardiovascular subjects. Alirocumab reduced all-cause mortality and serious adverse events, while evolocumab lowered the risk of MI [15]. An observational Polish study observed the safety and efficacy of evolocumab and alirocumab in heterozygous familial hypercholesterolemia (FH) patients after 3 months and 1 year of treatment. PCSK9 inhibitors significantly reduced LDL-C by 65 ± 14% after 3 months. Adverse effects like injection site reactions, flu-like symptoms, musculoskeletal symptoms, and fatigue were observed in 13%, 11.1%, 5.6%, and 5.6% of patients, respectively. The study was still observational, even after the design was carefully implemented and the data were thoroughly analyzed. The minimal number of patients constituted another constraint. This resulted from the therapeutic program’s tight inclusion requirements [16].

PCSK-9 inhibitors provide an extra LDL-C decrease when combined with statins and ezetimibe. The ODYSSEY outcomes trial studied the effect of adding alirocumab to statins at a high intensity in order to attain the LDL-C target in ACS patients. For comparative purposes, the estimated attainment using ezetimibe was also studied. The study involved 18,924 patients (9462 in the alirocumab group, 9462 in the control group), with a median 2.3 mmol/L LDL-C level at the baseline. A total of 88.8% of the patients received either atorvastatin at 40−80 mg/day or rosuvastatin at 20−40 mg/day, as a high-intensity statin treatment. A total of 94.6% of patients treated with alirocumab attained the LDL-C target (<1.4 mmol/L) compared to 17.3% in the control group. A total of 85.2% of the alirocumab group achieved an LDL-C < 1 mmol/L in patients with experience of a previous cardiovascular incident during a period of two years versus 3.5% in the control group. Based on the assumption that adding ezetimibe would reduce the LDL-C by 18%, the study estimated that adding ezetimibe would have reached the LDL-C aim of <1.4 mmol/L in 10.6% compared to <1 mmol/L in 0% of the patients. However, this assumption should not be translated into clinical practice, due to the possibility of an overestimation of the effect of ezetimibe [17].

A Chinese multicenter observational study compared the effect of PCSK-9 inhibitors combined with statins versus statins in very high risk ASCVD patients who had been undergoing a PCI for 6 months. The level of LDL-C decreased by 30.81% in the statins group and in the PCSK-9 inhibitor patients by 42.57%. The percentage of participants with an LDL-C ≤ 1.4 mmol/L was significantly increased from 2.99% to 18.43% in the statins-based group and, in the PCSK-9 inhibitor patients, from 10.36% to 47.69%. Moreover, an LDL-C ≤ 1.0 mmol/L was significantly increased from 0.23% to 6.11% in the statins-based group and, in the PCSK-9 inhibitor patients, from 5.29% to 29.26%. In terms of the reduction in the risk of MACEs, no significant difference was observed between the both groups. Being an observational study of real-world PCI patients, this study has various limitations. First, certain potential confounding factors may still remain that have not been taken into account, even though the full adjusted Cox model and propensity score weighting were established to eliminate them. Second, PSCK-9 inhibitors were only used for a month in the actual world settings due to financial constraints. Thus, the study was unable to determine how long-term PSCK-9 inhibitor medication affected the clinical results and the lipid-lowering effectiveness in individuals who had been subject to a PCI [18].

A network meta-analysis of 83,660 individuals was conducted involving adults taking the maximum tolerated statin dose or statin-intolerant patients to compare the effect of ezetimibe or PCSK9 inhibitors on cardiovascular outcomes. Adding ezetimibe or PCSK9 inhibitors to statins had no effect on MI or stroke in moderate and low cardiovascular risk patients. But they significantly reduced stroke and non-fatal MIs in subjects with a high or very high cardiovascular risk [19].

Summarizing the benefits and risks of lipid-lowering drugs, particularly for individuals with different levels of CVD risk, is crucial for guiding treatment recommendations. A meta-analysis comprising 237,870 subjects analyzed the benefits of statins, ezetimibe, and PCSK9 inhibitors in decreasing MACEs, using the number needed to treat (NNT). Statins significantly reduced MACEs compared to ezetimibe and PCSK9 inhibitors, with an NNT of 31, 18, and 18, respectively. No significant difference was found in terms of safety between statins and ezetimibe; however, there were reactions at the injection site when using PCSK9 inhibitors [20].

A SwissDiab observational study of two hundred and ninety-four patients with DM2, who did not meet the LDL-C target as per the European guidelines, were subjected to intensified lipid-lowering treatments and their effect on MACEs was investigated. The proportion of patients that attained the LDL-C target was 13.3% with the high-intensity statins, 27.9% with ezetimibe, 53.7% with PCSK9i inhibitors, 3.1% with both ezetimibe and PCSK9i inhibitors, and 1.7% did not reach the target. The study’s foundation based on a set of theoretical presumptions is a significant limitation. The research may exaggerate the impact of up-titrating low- and medium-intensity statins on LDL-C levels, and it fails to account for the possibility that certain patients may not be able to receive high-intensity statin medication due to statin intolerance. The amount of LDL-C reduction that could be achieved by optimizing statin therapy has probably been overestimated. This, in turn, would lead to an understatement in regard the amount of additional intensifications required in regard to the current lipid-lowering medication to guarantee that patients meet the 2016 and 2019 LDL-C targets, respectively. The projected lipid-lowering effect of combination treatments is most likely the same [21].

LDL-C levels remain inadequately controlled in US adults with DM among people with ASCVD. Only 18.2% patients were treated with high-intensity statins, 5.1% used ezetimibe, and 0.6% were treated with PCSK9 inhibitors. Moreover, 21.1% of patients had an LDL-C < 70 mg/dL. It is worthy to note that within minority groups, ezetimibe and PCSK9 inhibitor utilization were highest among non-Hispanic White populations [50].

### 2.4. Bempoedic Acid

Recently, bempedoic acid was authorized for the treatment of lipids in patients with heterozygous familial hypercholesterolemia (HeFH) or ASCVDs. By blocking adenosine triphosphate-citrate lyase (ACL) and, hence, cholesterol production, bempedoic acid increases the expression of LDL receptors and the plasma clearance of LDL-C. It is an intracellularly activated prodrug that is taken orally. It is not present in muscle or adipose tissue cells, but it is activated in liver and kidney cells to a lesser degree. Consequently, it has a much lower potential for myotoxicity than statins. Because of its distinct action, bempedoic acid can be used in combination therapy with other lipid-lowering medications [51].

Laufs et al. evaluated the safety and effectiveness of bempedoic acid in patients who were not taking statins. The LDL-C decreased by 26.5%, as a percentage, between the baseline and at 12 weeks of treatment [52]. The percentage decrease in LDL-C from the baseline to 12 weeks of treatment was 15.4% in a study by Goldberg et al., which had a sample size of 779 and added bempedoic acid to the maximally tolerated statin medication [53].

The negative effects on muscles are lower in all of the bempedoic acid patients that are included in this study than in the control group. Bempedoic acid lowers blood levels of LDL-C, boosts the liver’s absorption of LDL particles, and inhibits ATP citrate lyase. This prodrug needs acyl-CoA-synthetase-1, which is exclusively found in the liver, to activate it. Therefore, even though statins and bempedoic acid function via the same mechanism, bempedoic acid has fewer muscle-related side effects, such as myopathy and rhabdomyolysis [54]. Therefore, bempedoic acid may be a good substitute for statin-intolerant individuals. A systematic review assessed the effectiveness of recently approved medications for dyslipidemia, viz. bempedoic acid, evolocumab, alirocumab, and inclisiran, in lowering LDL-C. With bempedoic acid 216 (10%), more severe adverse events were recorded, including ovarian adenoma, hepatic cancer, and myocardial infarction [55]. The addition of patients with ASCVD or a bigger sample size (n = 2230) may be the cause of this. Additionally, bempedoic acid is administered orally, which raises the possibility of side effects. 

### 2.5. Inclisiran

Inclisiran is a synthesized small interfering RNA. It antagonizes PCSK9 mRNA and inhibits PCSK9 synthesis, which is a serine protease that binds to the LDL receptor present on the liver cell surface, which degrades LDL-C, hence the increase in the blood LDL-C concentration (Figure 1) [56,57]. It was approved to be marketed in EU in 2020 and by the FDA and Singapore Health Sciences Authority in 2021. It is administered subcutaneously, as a single dose on day one, then after three months, and then every six months, due to its long half-life [58,59].

In the ORION-9 trial, inclisiran caused a 47.9% reduction in LDL-C at day 510 [22]. In the ORION-10, and ORION-11 trials, at month 17, inclisiran, on top of the maximum tolerated statin dose, significantly reduced levels of LDL-C from 49.9% to 52.2%. Between months 3 and 18, it decreased the time-adjusted LDL-C from 49.2% to 53.8% compared to the placebo [23]. In the ORION-18 trial, involving a large trial in Asia, inclisiran significantly reduced the LDL-C by 57.2% when given with statins, with no side effects [24]. In the ORION-8 trial, with the largest (more than 12,000 patients) and longest follow-up (up to three more years) for patients from the ORION-3, ORION-9, ORION-10, and ORION-11 trials, inclisiran showed long-term safety, with constant and efficient lowering of LDL-C [60]. The VICTORION-INITIATE trial, an open-label, randomized, multicenter trial, studied the efficacy of the “Inclisiran First” execution strategy versus standard care on the levels of LDL-C in ASCVD patients and patients with LDL-C levels more than or equal to 70 mg/dL, even though they were receiving the maximum tolerated statin treatment. Inclisiran substantially decreased LDL-C by 60% from the baseline to day 330, while standard care only reduced LDL-C by 7.0%. Also, it substantially accomplished the LDL-C target compared to standard care [25]. The ORION-15 trial showed that at day 180, inclisiran was well-tolerated and achieved a statistically significant decrease in LDL-C by 65.3% and PCSK9 by 79.2%, over 12 months, in Japanese patients with hypercholesterolemia and HeFH [61].

Moreover, inclisiran led to a substantial and consistent decrease in LDL-C in glycemic patients from −47.6% to −51.9% and according to Body Mass Index strata from −48.8% to −54.4% at day 510, with transient mild-to-moderate adverse effects at the injection site [62].

Studies on the effect of inclisiran on reducing MACEs are still ongoing in the ORION-4, VICTORION-1 PREVENT and VICTORION-2 PREVENT trials.

The less frequent administration of inclisiran doses will improve patient compliance and, hence, will ensure treatment adherence, and this will eventually lead to improved LDL-C levels. When compared with evolocumab and alirocumab, inclisiran showed better patient adherence at 69.9% versus 48.6% and 51% for evolocumab and alirocumab, respectively [63].

## 3. Cost Effectiveness

Despite the use of statin therapy, patients with dyslipidemia and high cholesterol/triglyceride concentrations remain at significant risk of MACEs. To be widely adopted in clinical practice, new lipid-lowering therapies must both prevent MACEs and demonstrate cost effectiveness to ensure their widespread utilization by patients, physicians, and insurers. While non-statin LLTs have shown cardiovascular value, their cost effectiveness is controversial.

Decision makers in the healthcare industry, such as patients, physicians, hospitals, commercial health systems, and public payers, frequently face the difficult task of deciding which new or current interventions or programs to devote their meager resources to. Ideally, this decision is made after weighing the costs, risks, and health advantages of each option. When limited resources are available, a cost-effectiveness analysis can help decision makers make sense of the trade-offs between the costs, harms, and benefits of alternative treatments. It does this by combining these factors into a single metric, called the incremental cost-effectiveness ratio (ICER). An analytical tool, known as cost-effectiveness analysis, computes and presents the costs, benefits, and harms of an intervention (intervention A) and at least one alternative (intervention B) as a ratio of the incremental effect (effectiveness of intervention A–effectiveness of intervention B) and the incremental cost (cost of intervention A–cost of intervention B). The ICER is the name given to this ratio. The additional resources (such as medical care expenditure and costs from productivity changes) incurred when using intervention A over intervention B are represented by the incremental cost in the numerator. The additional health outcomes (such as the number of disease cases avoided or the quality-adjusted life years [QALYs] gained) that result from using intervention A over intervention B are represented by the incremental effect in the ICER’s denominator [64].

### 3.1. Statins

Low-cost statins are not only cost effective, but they also offer cost savings for most patients with even slightly increased cholesterol or any risk factors for coronary heart disease. The significant possible health benefits and cost savings to the healthcare system from preventing coronary heart disease events outweigh the possible adverse effects of long-term statin therapy [65]. In Scotland, the emergence of generics has made preventive statin treatment cost effective, enhancing the expansion of the authorization for statin treatment to guarantee that more patients who could possibly benefit from statins are treated. Absolute risk reduction-guided treatment is more attractive and economical for clinicians than 10-year risk scoring [66]. In a US randomized controlled trial of 20,536 patients, treatment with the generic simvastatin was found to be cost effective. The use of a daily dose of the generic simvastatin of 40 mg will create healthcare savings, even if it is only used for 5 years, due to the avoidance of vascular event hospitalizations that incur higher expenses [67]. In the contemporary UK population, a study estimated the overall health impact and cost effectiveness of statin treatment of various intensities across various population categories (age, sex, LDL-C, and cardiovascular risk). The lifetime statin treatment was very cost effective and increased the quality-of-life-adjusted survival in all the categories and across all people who were 40–70 years old. Higher intensity statin treatment was cost effective in subjects with increased LDL-C concentrations or at increased cardiovascular risk. The long-term use of statins needs to be enhanced in order to improve its health benefits. Restarting statin therapy in patients has been proven to be effective, but demands additional effort and resources. Statin use is much lower in low- and middle-income countries, due to the deficiency of in-depth cost-efficiency assessments. Its use in high-risk primary and secondary prevention populations was 2% and 8.2%, respectively, in low-income countries. In lower middle-income countries, its use was 6.3% and 21.2%, respectively, and 13.8% and 31.6%, respectively, in countries with an upper middle income [68].

Patients experiencing statin-associated adverse events incur higher medical costs compared to those with high adherence to statin therapy. Patients with SAAEs also face higher medical expenses compared to individuals with low statin adherence or those who discontinue statin treatment within the first year after a myocardial infarction [69]. These outcomes underscore the importance for health systems to allocate resources towards reintroducing statins to patients experiencing SAAEs and exploring alternative therapies.

### 3.2. Ezetimibe and/or PCSK9

A monotherapy of ezetimibe is a cost-effective alternative to statins in patients with high levels of LDL-C who cannot tolerate statins [70].

Adding ezetimibe to atorvastatin provides greater clinical benefits than fixed-dose atorvastatin or dose titration, although at an increased cost. The cost-effectiveness ratios support ezetimibe adoption by the Canadian healthcare system [71]. A study assessed the cost effectiveness of adding ezetimibe to statin treatment versus statin monotherapy from a US healthcare payer perspective. Ezetimibe combined with statins is a cost-effective strategy for CHD and stroke prevention, particularly in high-risk patients [72]. A simulation study assessed the economic viability of adding ezetimibe and PCSK9 inhibitors to statins for the secondary prevention of CVD, and it showed that adding ezetimibe is cost effective, while adding PCSK9 inhibitors, either alone or in combination, is not. For PCSK9 inhibitors, the ICER was USD 68,910 for every quality-adjusted life year (QALY); for ezetimibe, it was USD 20,242 per QALY; and, for both medications, it was USD 51,552 per QALY. The sensitivity analysis showed the robustness of the findings and indicated that the demographic characteristics had no effect on the ICER [73]. In the INFORCE study in the UK, changing to ezetimibe/simvastatin combination treatment versus doubling the submaximal tolerated statin doses, in patients with ACS, reduced the LDL-C by almost 30% and 4%, respectively; moreover, it was estimated to be more cost effective at <GBP14,000/QALY [74]. A Markov cohort model evaluated the cost effectiveness of statin versus combination lipid-lowering treatments for secondary cardiovascular prevention from the perspective of Germany’s healthcare system. Ezetimibe, evolocumab, and alirocumab provided varying degrees of patient benefit at different financial costs [75]. PCSK9 inhibitors require significant price reductions or prescribing restrictions to meet the cost-effectiveness criteria [76]. A systematic review analyzed 13 studies on the cost effectiveness of non-statin lipid-lowering treatments in T2DM patients, with or without cardiovascular disease. Ezetimibe was generally cost effective, while PCSK9 inhibitors showed limited cost effectiveness [77]. A Markov model was used to evaluate the cost effectiveness of PCSK9 inhibitors for coronary heart disease, ischemic stroke, and mortality in high-risk patients in Norway. The high cost of PCSK9 inhibitors limits their cost effectiveness, except for older, high-risk patients that need secondary prevention. Future price reductions could improve their value. Further research is required to assess their long-term preventive benefits [78]. The UK National Institute for Health and Care Excellence (NICE) conducted a single technology appraisal (STA) of evolocumab, evaluating its clinical and cost effectiveness as a single treatment or in combination with statins and/or ezetimibe for adult patients with primary hypercholesterolemia (including mixed dyslipidemia). This included individuals who did not attain optimal LDL-C levels with statins or could not tolerate them. Evolocumab was deemed cost effective for certain patient subgroups within the UK’s NHS, particularly in HeFH patients, but its broader adoption was limited by high ICERs [79]. A Markov modeled 1000 hypothetical patients, to be like the FOURIER trial, to evaluate the cost effectiveness of a PCSK9i and statin treatment approach relative to the statins-alone approach. The study measured the incidence of events, outcomes, health insurance, and the cost of care from the existing literature. The results showed that in order for PCSK9i to be an economically viable investment for insurers, higher discounts will be needed, with a price decrease of 62% to 83% to be cost effective, and in order to become economically sound for private payers [80]. A US simulation CVD Policy Model of adults who were aged 35 to 94 years evaluated the cost effectiveness of PCSK9 inhibitors compared to ezetimibe in patients with ASCVD or heterozygous FH. Over 5 years, the use of PCSK9 inhibitors was projected to decrease the cost of cardiovascular care by USD 29 billion; however, the drug costs increased by USD 592 billion. On the other hand, using statins in these populations was projected to save USD 12 billion. The use of PCSK9 inhibitors was not cost effective and was projected to substantially increase US healthcare costs [81]. Based on the viewpoint of the NHS, a study evaluated the cost effectiveness of statins in combination with ezetimibe, evolocumab, and alirocumab for secondary cardiovascular prevention. Ezetimibe was cost effective and discounts from 37% to 53% are need for PCSK9 inhibitors be cost effective. The findings held up well when subjected to probabilistic, scenario, and univariate sensitivity analysis [82].

### 3.3. Bempedoic Acid

From the standpoint of the US healthcare system, the cost effectiveness of a recently authorized combination of bempedoic acid and ezetimibe in patients with ASCVD was investigated. In the high-intensity statin group, bempedoic acid and ezetimibe’s ICER was USD 188,000/QALY; in the low/moderate-intensity statin group, it was USD 175,600/QALY (132,700–264,700; cost effective in 0% of 10,000 simulations). With a lifetime cost of USD 25,600 and an ICER of USD 92,600/QALY (66,000–152,100; cost effective in 59% simulations), bempedoic acid and ezetimibe generated 0.28 extra QALYs in the statin-free group [83]. With an acquisition cost of less than USD 600 per year, bempedoic acid might be economical for the Australian healthcare system. When compared to statin therapy alone, bempedoic acid was expected to save 103 (discounted) QALYs and 122 (discounted) years of life per 1000 people. In the Australian context, bempedoic acid would be deemed cost effective at an acquisition cost of AUD 584.40 per year (USD 397.01), with an incremental cost-effectiveness ratio of AUD 49,890 per QALY gained (USD 33,893) and AUD 42,433 for each year of life saved (USD 28,827) [84].

### 3.4. Inclisiran

In the Swiss healthcare system, inclisiran added to standard care lipid-lowering therapy could be cost effective and achieve major reductions in the cardiovascular disease burden depending on its price. It may be cost effective at a cost-acceptability threshold of CHF 30,000 if priced at CHF 500 per dose. A cost-acceptability threshold of CHF 250,000 is required if priced at CHF 3000 [85]. When inclisiran was used as an additive to the standard care therapy in Singapore it led to more therapeutic significant effects with an increase in costs, with an incremental cost-effectiveness ratio (ICER) of USD 43,853/QALY when compared to the standard of care treatment [86]. In China, the use of inclisiran when combined with statins compared with statins alone was not cost effective in dyslipidemic patients based on its used price (USD 2973.49/injection). In order to be economical, its price needs to be reduced by at least 88%. The robustness of the outcome was validated after all the model inputs were assessed across a broad range of values in the Markov model simulation’s sensitivity analysis [87]. In Australia, the cost of inclisiran should be reduced by 60% in order to be cost effective [88]. A UK Markov model adapted to Singapore, which included different populations viz. ASCVD, primary prevention with elevated risk, primary prevention heterozygous familial hypercholesterolemia, and secondary prevention heterozygous familial hypercholesterolemia, analyzed the cost effectiveness of inclisiran/standard care (statin, ezetimibe) versus standard care, evolocumab/standard care, and alirocumab/standard care. Inclisiran/standard care led to greater costs and QALYs when compared to standard care. Inclisiran/standard care was more effective and less costly when compared to evolocumab/standard care and alirocumab/standard care. The sensitivity and scenario evaluations revealed that the model’s uncertainties had no effect on the outcomes. The acquisition cost, effectiveness, and ratios of inclisiran, which convert LDL-C decreases into the risk of CV death, had the most impact on the model’s outcomes [89].

The effect of a novel drug on medical expenditures is identified by the targeted patients’ size, drug efficacy duration, price, and healthcare expenses. Since PCSK9 inhibitors are not only used for a small number of patients, but are also lifelong therapies, their high costs are challenging when compared with expensive drugs used for rare conditions for a short period, regardless of the cost reduction expected due to the prevention of ASCVD. Decreasing the price of PCSK9 inhibitors is challenging. Being a biological therapy, its manufacture is far more expensive than small drug molecules. Moreover, over the years, the price of biological therapies has increased. Even biosimilars are not expected to be more cost effective, due to the prolonging of the exclusive market rights of brands and the complexity involved in the manufacturing process. In the UK, PCSK9 inhibitors were only approved for use in NHS after companies made an additional price discount, even though its price was originally less than half the price that the UK’s NICE draft guidance recommends for new drugs for cholesterol disorders.

One strategy for controlling expenses is to restrict treatment with PCSK9 inhibitors to very high-risk patients and the detection of true statin-intolerant patients. Another strategy is to support medical initiatives that improve patients’ statin adherence within those patient populations at high risk of ASCVD, especially given that more than one third of patients with ASCVD and FH are not taking statins even though their cost effectiveness and efficacy have been proven. Patient adherence to statin therapy is estimated to save USD 12 billion over five years [65].

Elevated discontinuation levels were recorded for evolocumab and alirocumab compared to inclisiran, due to the required increased frequency of such injections. Also, even though the unit dose of inclisiran is more expensive than evolocumab and alirocumab, it has lower medicine acquisition costs, due to its lower administration frequency [89].

Despite guidelines and recommendations and the well-established benefits of LLT in preventing CVD, achieving treatment targets remains disappointing. A key barrier to optimal LLT is therapy discontinuation. Statins remain cost effective and safely reduce atherosclerotic CVD events. Ezetimibe costs more than statins, but is significantly cheaper than PCSK9 inhibitors. The availability of novel lipid-lowering agents has broadened the treatment possibilities, but access varies by region. PCSK9 inhibitors and ezetimibe have similar relative risk reductions, but the absolute benefits depend on individual cardiovascular risk. Most guidelines do not systematically evaluate the cardiovascular value of adding PCSK9 inhibitors for all risk groups. PCSK9 inhibitors have minimal adverse effects, but face economic challenges, with high costs and limited availability in low-income countries; moreover, it requires subcutaneous administration. Ezetimibe is more affordable and orally administered, making it a preferred first add-on option. Cost-effectiveness analyses show variable results, but in very high-risk patients, PCSK9 inhibitors may meet cost-effectiveness thresholds in select populations. A patient-centered approach, emphasizing adherence and personalized treatment regimens, is critical for improving outcomes. Intensive LDL-C reduction should start early and be maintained over time. Treatment recommendations should be adaptable, based on drug availability, reimbursement policies, and patient preferences. Optimizing LLT access and encouraging personalized treatment strategies will significantly reduce the burden of CVD. This emphasizes that no single approach or combination of approaches guarantees improved cardiovascular outcomes and the guidelines available should be more clinically feasible. Instead, a patient-tailored approach should be adopted to optimize treatment effectiveness. This highlights the importance of lipid clinics; in lipid clinic patients, LDL-C target achievement significantly improved from 14.6% to 41.7% without the use of PCSK9 inhibitors, compared to an increase from 21.4% to 33% in standard care patients [90].

## 4. Conclusions

Managing modifiable risk factors, particularly LDL-C, remains a cornerstone of CVD prevention. While statins are the primary therapy, additional lipid-lowering agents like ezetimibe and PCSK9 inhibitors provide further options for high-risk patients. While international guidelines provide broad recommendations, they vary in regard to treatment targets and approaches, necessitating a more individualized, evidence-based application in clinical practice. Shared decision making is essential in determining treatment strategies, balancing clinical benefits, patient preferences, and economic considerations. As lipid-lowering therapies continue to evolve, adherence to evidence-based guidelines will be critical in optimizing cardiovascular health and reducing the disease burden.

## Figures and Tables

**Table 1:** Studies on LCL-C-lowering treatments in various populations.

Study	Population	Number of Participants	Treatment	Control	Effect	Ref.
RACING	ASCVD	3780	10 mg R + 10 mg E	20 mg R	R/E was non-inferior to the high dose R.	[5]
Meta-analysis	ASCVD	Over 5000	LIS–MIS/E	HIS	Combined LIS–MIS/E substantially decreased LDL-C, no significant difference in total cholesterol, HDL-C, triglyceride, Apo A1 high-sensitivity C-reactive protein, or Apo B.	[6]
Comparative cohort, Korean, observational study	CVD	3,390,305 adults	LIS–MIS/E	HIS	Combined treatment significantly decreased the risk of a composite outcome, individual MIs and stroke with a HR of 0.84, 0.81, and 0.78, respectively. No difference in all-cause mortality.	[7]
Observational study	ACS patients after percutaneous coronary intervention	286,817	MIS/E	HIS	In the HIS group, the primary outcome (ischemic stroke, MI, and all-cause mortality) was 4.73 events/100 person/year, and in the MIS/E group, it was 4.29 events/100 person/year. The risk of MI: no difference between both treatments.	[8]
Observational study	Patients who underwent a PCI	45,501	MIS/E	HIS	MIS/E was similar to HIS in terms of long-term risk decrease in MACEs. Combination treatment was correlated with a substantial decrease in the occurrence of new-onset DM (12.5% in the case of the monotherapy and 10.7% in the case of the combination therapy).	[9]
Meta-analysis	Patients at the time of ACS	20,291	HIS/E	Placebo	Addition of ezetimibe significantly reduced LDL-C at 7 days (−19.55 mg/dL), 1 month (−24.67 mg/dL), 3 months (−18.01 mg/dL), and 10–12 months (−16.90 mg/dL) of treatment. It also significantly reduced total cholesterol at 7 days (−21.05 mg/dL), 1 month (−25.56 mg/dL), 3 months (−22.54 mg/dL), and 12 months (−19.68 mg/dL) of treatment. Death due to any reason, non-fatal stroke, ACS, ischemic stroke, and non-fatal MIs were substantially reduced.	[10]
Korean, phase IV, multicenter, randomized controlled trial	ASCVD	270	R and E combination therapy, 10/10	R 20 mg	After treatment for 12 and 24 weeks, the dual treatment significantly decreased LDL-C compared to the high-intensity statin therapy (−22.9% for the combination therapy, −15.6% for the monotherapy, after 12 weeks; and −24.2% for the combination therapy, −12.9% for the monotherapy, after 24 weeks).	[11]
Korean, phase III, multicenter, double-blind, randomized study	Primary hypercholesterolemia patients	422	P/E	P	Combination therapy substantially decreased LDL-C by >50%	[12]
ODYSSEY outcomes trial	ACS	18,924	Alirocumab	Placebo	Alirocumab substantially decreased lipoprotein(a) and LDL-C leading to a 15% reduction in MACEs.	[13]
Retrospective cohort study	ACS patients who underwent a PCI	1564	Evolocumab	With high LDL-C (≥3.2 mmol/L without statin treatment, ≥2.3 mmol/L after taking LIS or MIS, or ≥1.8 mmol/L after taking HIS for ≥4 weeks)	Addition of evolocumab to statins decreased LDL-C by 42.48%.	[14]
Meta-analysis	High-risk cardiovascular patients	54,311	PCSK9 inhibitors	Placebo	Alirocumab reduced all-cause mortality and serious adverse events, while evolocumab reduced the risk of MIs.	[15]
Observational polish study	Heterozygous familial hypercholesterolemia	55	Alirocumab or evolocumab	43 (HIS), 10 (maximum tolerated dose of statins), 2 (no treatment)	PCSK9 inhibitors significantly reduced LDL-C by 65 ± 14% after 3 months.	[16]
ODYSSEY outcomes trial	ACS patients	18,924	Adding alirocumab to HIS	A 40−80 mg daily or R 20−40 mg daily as HIS treatment	A total of 94.6% of patients treated with alirocumab attained the LDL-C goal, which was less than 1.4 mmol/L compared to 17.3% in the control group. A total of 85.2% of the alirocumab group achieved LDL-C less than 1 mmol/L in subjects who had experienced a prior cardiovascular incident during two years versus 3.5% in the control group.	[17]
Chinese multicenter observational study	3063 patients	Very high risk ASCVD patients who underwent a PCI	PCSK-9 inhibitors/S	S	The level of LDL-C decreased by 30.81% in the S group and in the PCSK-9 inhibitor subjects by 42.57%. The percentage of participants with LDL-C ≤ 1.4 mmol/L was significantly increased from 2.99% to 18.43% in the S subjects and from 10.36% to 47.69% in the PCSK-9 inhibitor subjects. Moreover, LDL-C ≤ 1.0 mmol/L was significantly increased from 0.23% to 6.11% in the S subjects and from 5.29% to 29.26% in the PCSK-9 inhibitor subjects.	[18]
Meta-analysis	Cardiovascular outcomes	83,660	Adding E or PCSK9 inhibitor to S	Statins	Adding E or PCSK9 inhibitor to S had no effect on MIs or stroke in moderate and low cardiovascular risk patients. But they significantly reduced the occurrence of stroke and non-fatal MIs in subjects with a high or very high cardiovascular risk.	[19]
Meta-analysis	MACE	237,870	S, E, and PCSK9 inhibitors	Placebo	S significantly reduced MACEs compared to E and PCSK9 inhibitors with an NNT of 31, 18, and 18, respectively.	[20]
A SwissDiab observational study	Patients with DM2 who failed the LDL-C target	294	HIS; E; PCSK9i; both E and PCSK9i	Placebo	The proportion of patients that attained the target: 13.3% with HIS, 27.9% with E, 53.7% with PCSK9i, 3.1% with both E and PCSK9i, and 1.7% did not reach the target.	[21]
ORION-9 trial	Familial hypercholesterolemia	482	Inclisiran	Placebo	A total reduction of 47.9% in LDL-C at day 510.	[22]
ORION-10 ORION-11	ASCVD	1561	Inclisiran on top of the maximum statin dose	Placebo	Reduced the levels of LDL-C by 49.9% to 52.2%.	[23]
ORION-18	ASCVD	345	Inclisiran	Placebo	Reduced LDL-C by 57.2% when given with statins, with no side effects.	[24]
VICTORION-INITIATE trial	ASCVD; subjects having LDL-C concentration ≥ 70 mg/dL, even though they received the maximum tolerated statin treatment	450	Inclisiran	Placebo	Decreased LDL-C by 60% from the baseline to day 330, while standard care only reduced LDL-C by 7.0%.	[25]

ASCVD: atherosclerotic cardiovascular disease; A: atorvastatin; R: rosuvastatin; E: ezetimibe; LIS: low-intensity statin; MIS: moderate-intensity statin; HIS: high-intensity statin; S: statin; P: pitavastatin; MI: myocardial infarction; NNT: number needed to treat; PCSK9: proprotein convertase subtilisin/kexin type 9.
